# Protecting HaCaT cells from ionizing radiation using persimmon tannin-*Aloe* gel composite

**DOI:** 10.1080/13880209.2020.1767158

**Published:** 2020-06-01

**Authors:** Xi Qian, Zhongmin Wang, Jinliang Ning, Chaoke Qin, Lin Gao, Na He, Dahong Lin, Zhide Zhou, Guiyin Li

**Affiliations:** a School of Materials Science and Engineering, Guilin University of Electronic Technology, Guilin, China; b School of Life and Environmental Sciences, Guilin University of Electronic Technology, Guilin, China; c China Nonferrous Metal (Guilin) Geology for Mineral Co., Ltd, Guilin, China

**Keywords:** Human epidermal keratinocytes cells, Radiation resistance, X-rays

## Abstract

**Context:**

Persimmon tannin (extract of *Diospyros kaki* L.f [Ebenaceae]) and *Aloe* gel **(**extract of *Aloe vera* (L.) Burm.f. [Asphodelaceae]) are known as anti-radiation agents. However, radiation resistance of the persimmon tannin-*Aloe* gel composite remains inconclusive.

**Objective:**

To investigate the capacity of the persimmon tannin-*Aloe* gel composite to protect against ionising radiation at the cellular level.

**Materials and methods:**

HaCaT (human epidermal keratinocytes) cells were pre-treated with PT-A-1 (the mass ratio of persimmon tannin and *Aloe* gel was 2:1) or the single component (persimmon tannin or *Aloe* gel) at various concentrations (0, 50, 100, 200, 400, 800 μg/mL. Control group: medium with no HaCaT cells), and then radiated with X-rays (radiation dose: 4, 8, 12, 16, and 20 Gy). Cell viability, cell apoptosis, and radiation-induced intracellular reactive oxygen species (ROS) generation were analysed by CCK-8, Hoechst 33258 staining/flow cytometry, and 2′,7′-dichlorfluorescein diacetate (DCFH-DA) assay, respectively, for 12 or 24 h incubation after radiation.

**Results:**

The optimal radiation dose and post-radiation incubation period were determined to be 8 Gy and 12 h. CCK-8 activity detection showed that the cell activity was 77.85% (*p* < 0.05, IC_50_ = 55.67 μg/mL). The apoptotic rate was the lowest (4.32%) at 200 μg/mL of PT-A-1 towards HaCaT cells. ROS production was the most effectively suppressed by 200 μg/mL PT-A-1 towards HaCaT cells.

**Discussion and conclusions:**

The persimmon tannin-*Aloe* gel composite has good radioprotective effect, and which will facilitate its clinic application as a potential natural anti-radiation agent in future.

## Introduction

At present, the problem of ionizing radiation pollution in China is increasing the incidence of ionizing radiation in the human body, which will lead to radiation diseases that are extremely harmful to human health (Burmeister 2000). Therefore, it is necessary to find better anti-radiation agents to prevent and control ionizing radiation pollution.

When ionizing radiation enters the human body, anti-radiation agents can be used to eliminate the threat. Anti-radiation agents are mainly divided into non-natural and natural anti-radiation agents. Non-natural anti-radiation compounds, such as sulfhydryl compounds, nitroxide, dibenzimidazole, superoxide dismutase, etc., can result in significant side effects on the human body. Natural anti-radiation compounds mainly include vitamins, melatonin, flavonoids, and anti-radiation herbaceous plants (Tang and Loganovsky 2018). Anti-radiation herbaceous plants reduce and eliminate the harm of ionizing radiation in the human body by blocking the related harmful reactions caused by free radicals generated by radiation, reducing DNA damage, and inhibiting apoptosis (Rostami et al. 2016).

Tannin, also known as tannic acid, is widely found in many natural plants such as persimmons (*Diospyros kaki* L.f [Ebenaceae]), tea leaves, ginkgo leaves, and grape seeds. It has a complex polyphenolic hydroxyl structure that is soluble in water and some organic solutions. Nowadays, Guangxi produces the most persimmons in China (Wang et al. 2017; Li et al. 2019). In order to ensure that most persimmons can mature normally, some green persimmons must be removed. Numerous studies have shown that green persimmons contain large amounts of persimmon tannin (Zhou et al. 2015; Ying et al. 2017). Persimmon tannin has proven useful for the prevention and treatment of ionizing radiation pollution (Wang et al. 2017; Wu et al. 2017; Li et al. 2018). Wu et al. (2015) studied the protective effect of tannin acid on human megakaryocyte injury induced by ionizing radiation. By pretreating human megakaryocytes with different concentrations of tannin, it was shown that tannic acid provided effective protection after 10 Gy ^60^Co γ-ray irradiation. Recently, our group indicated that persimmon tannin offered a potent radioprotective effect on cell vitality and cell apoptosis of γ-radiation exposure in HEK 293 T cells (Zhou et al. 2016).

*Aloe vera* (L.) Burm.f. (Asphodelaceae) contains over 70 components, including anthraquinones, vitamins, nonessential amino acids, essential amino acids, and inorganic compounds. It is reported that *Aloe vera* can ameliorate adverse radiation events on the epidermis and dermis owing to its ability to increase wound oxygenation and minimize the amount of dead tissue at radiation sites (Farrugia et al. 2019). Bala et al. (2018) found that *Aloe vera* gel extract reduced hepatic and renal damage, and that it ameliorated genetic damage as indicated by reduced chromosomal aberrations and apoptosis.

Human epidermal keratinocytes (HaCaT) are normal human epidermal cells that derived from the skin of human beings. In most cases, human skin is the first component of the human body damaged by ionizing radiation. Therefore, HaCaT cells are widely universal as radiation receptor cells, and can be used in experiments studying ionizing radiation protection materials and their effectiveness in protecting normal human cells (Chen et al. 2019).

In this study, the capacity of the persimmon tannin-*Aloe* gel composite to protect against ionizing radiation towards HaCaT cells was investigated. CCK-8 assay, flow cytometry, Hoechst 33258 staining, and DCFH-DA assay were used to evaluate the cell viability, apoptosis and intracellular reactive oxygen species (ROS) production after radiation.

## Materials and methods

### Materials and instruments

Persimmon tannin freeze-dried powder used in this experiment was obtained from Guangxi Huikun Co., Ltd (Guangxi, China). The persimmon tannin was extracted from green Gongcheng persimmons (Guangxi, China), then dried by the freeze-drying technique (JDG-0.2, Lanzhou Kejin Vacuum Freeze Drying Technology Co., Ltd (Lanzhou, China) to obtain the persimmon tannin freeze-dried powder. *Aloe vera* gel freeze-dried powder (200:1) was purchased by Guangdong Yuanlv Biological Engineering Co., Ltd (Guangdong, China). Human immortalized epithelial cells (HaCaT) were purchased from the cell bank of Peking Union Medical College Hospital (Beijing, China). Low endotoxin foetal bovine serum was provided by Zhejiang Tianhang Biotechnology Co., Ltd (Zhejiang, China), DMEM high glucose medium and trypsin were provided by Thermo Fisher Scientific (Waltham, MA, USA), 2′,7′-dichlorfluorescein diacetate (DCFH-DA) was provided by Sigma-Aldrich Co., Ltd. (St. Louis, MO, USA). Other chemical reagents were all biochemical grade.

Experimental instruments included a TU-1901 UV-visible spectrophotometer provided by Beijing Persee General Instrument Co., Ltd. (Beijing, China), a flow cytometer provided by Beckman Coulter, Inc. (Brea, CA, USA).

### Determination of active ingredients in persimmon powder

First, a 10% Lowry solution and 7.5% sodium carbonate solution were prepared. Then, in amounts of 0, 1.0, 2.0, 3.0, 4.0, and 5.0 mL, standard solutions of 1000 μg/mL gallic acid were added to a 100 mL volumetric flask, for use in the working fluid. Next, to prepare the persimmon powder solution, 0.1 g of persimmon powder was added to a 100 mL volumetric flask; 2.5 mL of persimmon powder solution was then pipetted into a 50 mL volumetric flask, and ultrapure water was added up to the mark line to prepare the test solution. Finally, a UV spectrophotometer was used to measure the absorbance to obtain a standard curve (Chavan and Singhal 2013). The assay was performed according to the gallic acid standard curve (y = 0.01194x + 0.0033, R^2^ = 0.9996). The content of persimmon tannin can be calculated according to the standard linear equation of gallic acid. According to the calculation, the persimmon tannin content in the persimmon freeze-dried powder used in the experiment was 25.00%.

### Determination of active ingredients in *Aloe* gel freeze-dried powder

*O*-Acetyl groups can be used as a calibration standard to quantitatively measure the content of the active ingredient in aloe lyophilized powder. Thus, the content of the active ingredient in an aloe lyophilized powder can be determined by measuring the *O*-acetyl group. The *O*-acetyl content in the aloe freeze-dried powder was determined by spectrophotometry and the corresponding standard curve was drawn. The prepared standard curve was used to determine the *O*-acetyl content in the aloe freeze-dried powder used in the experiment (Nazeam et al. 2017; Kumar and Kumar 2019). The standard curve equation for *O*-acetyl content is y = 2.2633x + 0.0125, R^2^ = 0.9995. Using the standard linear equation, the *O*-acetyl content in the aloe freeze-dried powder was calculated to be 0.60%.

### Preparation of persimmon tannin-*Aloe* gel composite

The persimmon tannin-*Aloe* gel composite was prepared according to the proportions of the active ingredients in the two materials. First, PT-A-1 composite, in which the mass ratio of persimmon tannin and *Aloe* gel was 2:1, was produced through a crosslinking reaction with glutaraldehyde. The precipitated composite material was then collected and placed in a vacuum drying oven at 50 °C for 6 h. After the composite was completely dried, it was pulverized to obtain the PT-A-1 composite powder.

In the same way, PT-A-2 composite, the mass ratio of persimmon tannin and *Aloe* gel was 1:1, was prepared according to the above method.

### Culture of HaCaT cells

The frozen HaCaT cells were quickly thawed in warm water at 37 °C; the thawed culture solution was aspirated (dropped into the thawed culture solution) into a 15 mL tube; the prepared foetal bovine serum content was 10%. Fresh high-sugar DMEM medium (2 mL), the frozen stock solution remaining in the HaCaT cells, was removed by centrifugation. The old medium in the centrifuge tube was removed after centrifugation, and the cells in the bottom of the centrifuge tube were blown upward by fresh medium to prevent further gathering (Abernathy et al. 2015). After the cell suspension was added to a 25 cm^2^ cell culture flask, the flask was placed in a thermostatic cell culture incubator at a constant temperature of 37 °C and a constant carbon dioxide content of 5%. The cells were cultured until the cell growth area accounted for 80–90% of the bottom of the cell bottle; afterward, the old medium was removed and trypsin-digested cells were added, and then the cells were blown downward by adding fresh medium, followed by centrifugation. The subsequent steps were the same as those described previously, and a heavy cell culture will result. The suspended cell fluid was added to two new cell culture flasks to complete the cell passage, and the cells were cultured repeatedly until the cell volume reached 80–90% (Hillman et al. 2011).

### CCK-8 assay of the effect of PT-A composite

The activity of the cells after irradiation experiments was measured via a CCK-8 assay (Painuli and Kumar 2016; Wu et al. 2017; Zhao et al. 2017). Firstly, after the synchronization of the HaCaT cells was complete, the old medium of each well was removed, and 90 μL of fresh foetal sugar medium with 10% foetal bovine serum content and 10 μL PT-A-1 (PT-A-2, PT, *Aloe* gel) was added. Subgroups with concentrations of 0, 50, 100, 200, 400, and 800 μg/mL were created. In addition, the blank group was set to medium only and no cells were seeded. Secondly, the cells of each experimental group were exposed to different X-ray doses after 24 h of culture, which were 4 Gy, 8 Gy, 12 Gy, 16 Gy, and 20 Gy. A control group was also needed. Thirdly, after irradiation, the old media in the wells of the cell plate were removed after 12 and 24 h incubation, respectively, and each cell culture well was gently washed three times with a sterile PBS solution. Fourthly, a mixture of 10 μL of CCK-8 and 90 μL of foetal high blood sugar medium containing 10% foetal calf serum was added to each experimental cell well. Finally, after the CCK-8 solution was completely activated within 3 h, and the cell plate was placed under a multi-plate reader to measure the absorbance (OD) of each well at 490 nm. The cell vitality was calculated as following Equation (1).

(1) $$\text{Cell}\;\text{vitality}\;(\%)=(\frac{OD_{\text{drug}}-OD_{\text{blank}}}{OD_{\text{control}}-OD_{\text{blank}}})\times 100\%$$

### Hoechst 33258 staining for detection of apoptosis

When cells are exposed to unfavourable external factors such as toxicity or irradiation, the process of apoptosis occurs, in which cells die naturally in order to maintain the internal stability of the body. The most common method of directly observing the characterization of apoptosis is to fluorescently stain cells with Hoechst 33258 (Kabir and Kumar 2016; Zhou et al. 2018). By observing the colour intensity of the fluorescent dye, cell apoptosis can be detected.

When the HaCaT cells were completely synchronized, the old medium in each well was removed. Subgroups with PT-A-1 (PT, *Aloe* gel) concentrations of 0, 50, 100, 200, 400, and 800 μg/mL were provided. In addition, the blank group was set only for the medium and was not seeded. After 24 h of culture, the HaCaT cells received the optimal X-ray radiation dose; meanwhile, a control group was needed. The cell plates used as the control group did not receive irradiation with X-rays. The HaCaT cells were irradiated and placed in the incubator. They were then cultured for 12 h; afterward, the cell fixative in each well was cleared, and each well was washed twice with sterile PBS for 3 min. Finally, 500 μL of Hoechst 33258 fluorescent dye was added to each well, and the cells were fully stained for 5 min. The fluorescent dye in each well was then removed, and each well was washed twice with PBS for 3 min. One drop of fluorescence quenching was added to each well; subsequently, the cell staining was observed using a 200-fold inverted fluorescence microscope, and a fluorescent staining photo was taken.

### Flow cytometry to detect apoptosis

Apoptosis is one of the common states of cells in the body (Yi et al. 2018), in this study, cell apoptosis was studied using an AnnexinV/FITC-PI apoptosis assay kit (Nattaporn et al. 2015). The cultured HaCaT cells were centrifuged; when the HaCaT cells were completely synchronized, the old medium in each well was removed. Subgroups with PT-A-1 (PT, *Aloe* gel) concentrations of 0, 50, 100, 200, 400, and 800 μg/mL were provided. In addition, the blank group was set only for the medium and was not seeded. After 24 h of culture, the HaCaT cells received the optimal X-ray radiation dose; meanwhile, a control group was needed. The control group only had 5 mL of medium and 5 mL of PBS solution, and no cells were inoculated. PBS was then poured into the centrifuge tube and 0.4 mL of Annexin V binding buffer was added. The cells were then resuspended at a final cell density of 1 × 10^6^ cells/mL of single cell suspension. Then, 5 μL of Annexin V-FITC was added to the cell suspension; the cells were removed and added to a flow tube, and apoptosis was detected by flow cytometry.

### Performance of persimmon tannin-*Aloe* gel composites in removing intracellular oxygen species

When the HaCaT cells were completely synchronized, the old medium of each well was removed, six subgroups for different PT-A-1 concentration with four parallel holes each were provided. After 24 h of incubation, the HaCaT cells were irradiated with an optimal dose of X-ray radiation. Meanwhile, the control group was set only for the medium and was not seeded. After being irradiated, the HaCaT cells were placed into the incubator for 12 h of cultivation. Then the medium was removed and each well was washed twice with PBS, and followed by addition of 10 μL of DCFH-DA solution and 90 μL of serum-free DMEM medium for 30 min at 37 °C. After incubation and being washed twice with PBS. Finally, the cell plate was placed into the multi-functional microplate reader at 480–525 nm detection in order to detect DCF fluorescence intensity, which was obtained according to the content of ROS (Martins et al. 2012).

### Statistical analysis

Each experiment was performed in triplicate. Data were expressed as the mean ± standard deviation (SD). All data in this study were analyzed with the SPSS 10.0 software package. A *p*-value <0.05 was considered statistically significant. All experiments were conducted in triplicate unless otherwise noted in the text.

## Results and discussion

### Content of active ingredients in persimmon tannin-*Aloe* gel composite

In this study, we use the standard curves of the gallic acid and the *O*-acetyl group to determine the contents of persimmon tannin and *O*-acetyl group in the persimmon-*Aloe* gel composite. For PT-A-1 composite, the contents of persimmon tannin and o-acetyl group were respectively calculated as 1.15% and 0.57%, thus the ratio was close to 2:1. Using the same method, we can determine that the contents of persimmon tannin and *O*-acetyl group in PT-A-2 composite were 0.585% and 0.586%, respectively, which is close to 1:1. Thus, the results showed that both proportions of the persimmon tannin-*Aloe* gel composite were prepared as expected.

### CCK-8 assay of the ionizing radiation protection ability of different component materials

The ionizing radiation protection abilities of different components were compared by CCK-8 assay. Tests analyzed the ionizing radiation protection provided by PT, *Aloe* gel, PT-A-1 composite and PT-A-2 composite. The radiation dose was 8 Gy and the incubation time was 12 h after irradiation.

**Figure 1.**
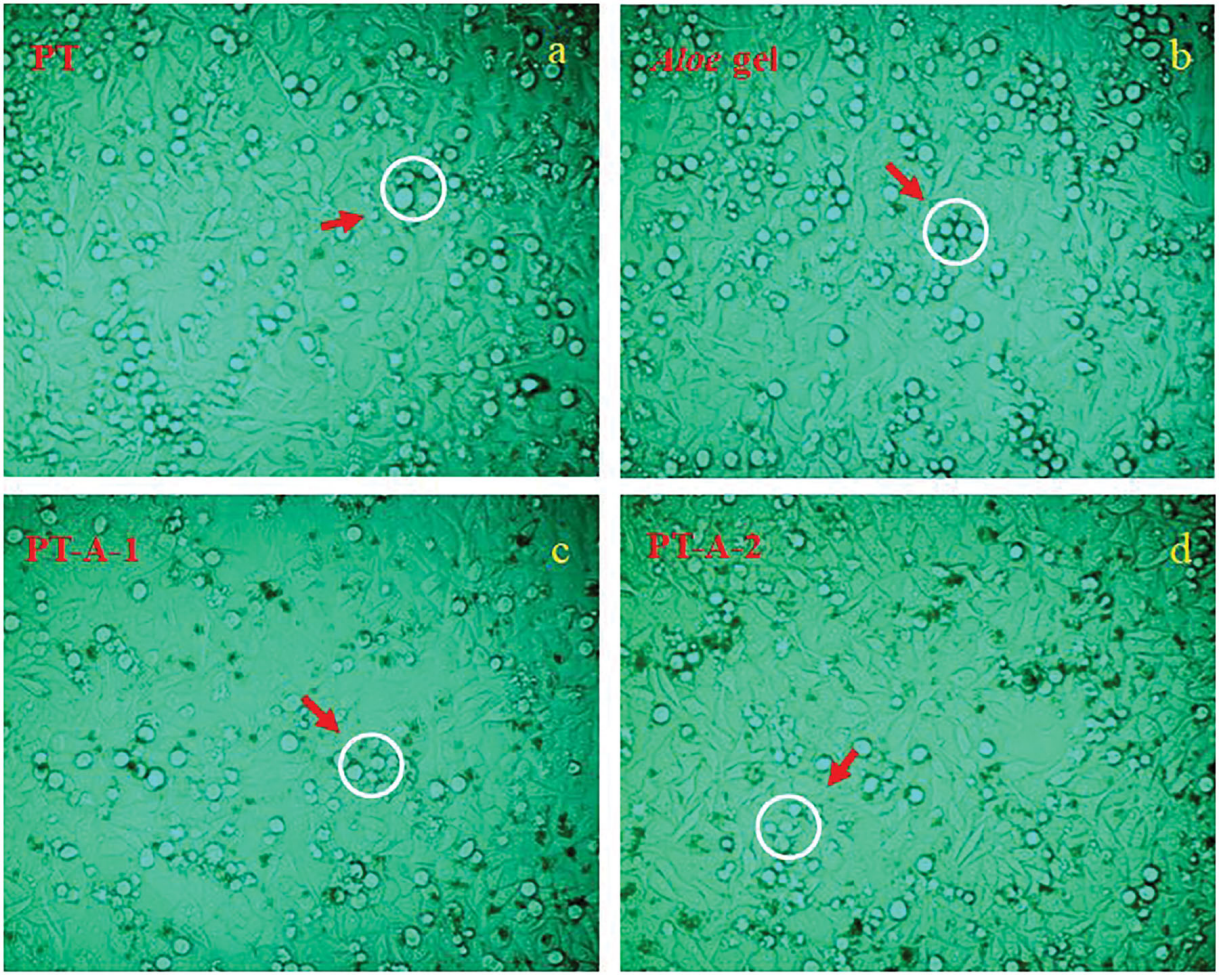
The HaCaT cells morphology with different ingredients at a concentration of 200 μg/mL after 8 Gy radiation pre-treatment for 12 h incubation (400×).

As shown in Table 1, after the irradiation test, the highest activity values among the cells pre-treated with the four components appeared in the experimental group with the pre-treatment concentration of 200 μg/mL. Additionally, it was shown that the radiation resistance of persimmon tannin-*Aloe* gel composite is superior to that of single materials. When the mass ratio of persimmon tannin and *Aloe* gel was 2:1 (i.e., PT-A-1), the HaCaT cells exhibited the highest degree of activity and the best radiation resistance. As shown in Figure 1, the cell morphology of each cell at 200 μg/mL was observed. Figure 1(c) shows the lowest amount of cell debris due to apoptosis. Therefore, PT-A-1 composite was selected as the optimal composite material for further investigation.

**Table 1.** Cell viability (%) of HaCaT cells with different ingredients after 8 Gy radiation pre-treatment for 12 h incubation.

Ingredient	Concentration					
	0 μg/mL	50 μg/mL	100 μg/mL	200 μg/mL	400 μg/mL	800 μg/mL
PT-A-1	48.592	59.915	70.246	77.849	67.561	55.884
PT-A-2	46.225	53.683	61.993	69.653	60.238	49.967
PT	48.627	51.528	56.209	62.871	57.891	46.261
*Aloe *gel	49.364	50.213	52.085	60.447	55.918	41.857

### CCK-8 assay of determination of optimal radiation dose and optimum concentration for PT-A-1 composite

**Figure 2.**
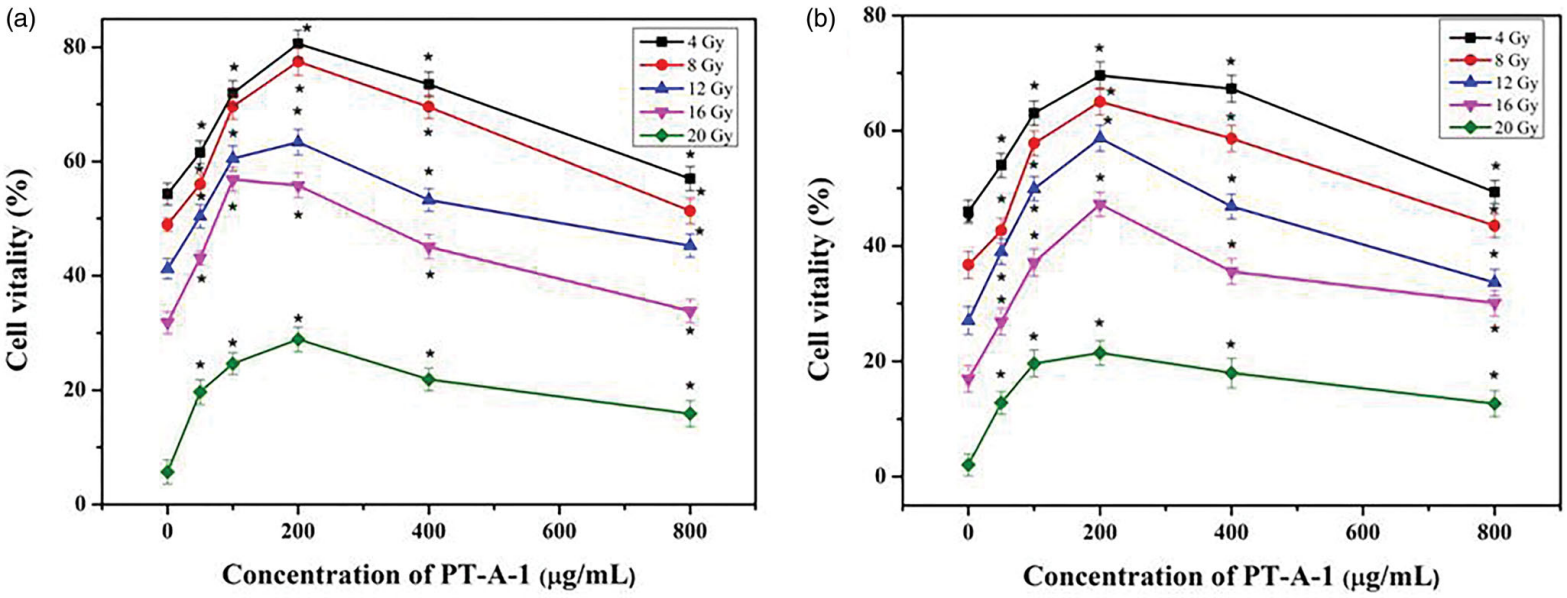
Cellular viability (%) of HaCaT cells with PT-A-1 for different concentrations and different radiation doses for 12 h incubation after X-ray irradiation (a); Cellular viability (%) of HaCaT cells with PT-A-1 for different concentrations and different radiation doses for 24 h incubation after X-ray irradiation (b). All above values are presented as the median from analysis of three independent experiments and the error bars indicate standard deviation (*n* = 3, *p* < 0.05). Symbol* represents *p* < 0.05 vs. blank group (Concentration of 0 g/mL).

The cell viability under both conditions was examined via a CCK-8 assay. As shown in Figure 2(a), the activity of HaCaT cells is continuously reduced as the dose of X-ray radiation increases, owing to the influence of X-ray irradiation. It can be seen that the activity of HaCaT cells first increased and then decreased with the increasing concentration of PT-A-1 composite. When the concentration of PT-A-1 composite reaches 200 μg/mL, HaCaT cell activity reached its maximum value. Among the five experimental groups, the group with the radiation dose of 8 Gy and a concentration of PT-A-1 composite that increased from 0 to 200 μg/mL exhibited the highest change of cell growth activity. When the dose of X-ray irradiation was 8 Gy, IC_50_ was calculated to be 55.67 μg/mL for PT-A-1 composite at 12 h incubation after irradiation. Therefore, a radiation dose of 8 Gy was selected in the subsequent experiment. As seen from Figure 2(a,b), the trend of results for 24 h was similar to the results of for 12 h incubation after X-ray irradiation. The cell activity at 12 h after irradiation was generally higher than that at 24 h after irradiation. In accordance with the above conclusions, a radiation dose of 8 Gy and a cell culture irradiation time of 12 h were selected as the optimal experimental conditions.

### Hoechst 33258 fluorescent staining

**Figure 3.**
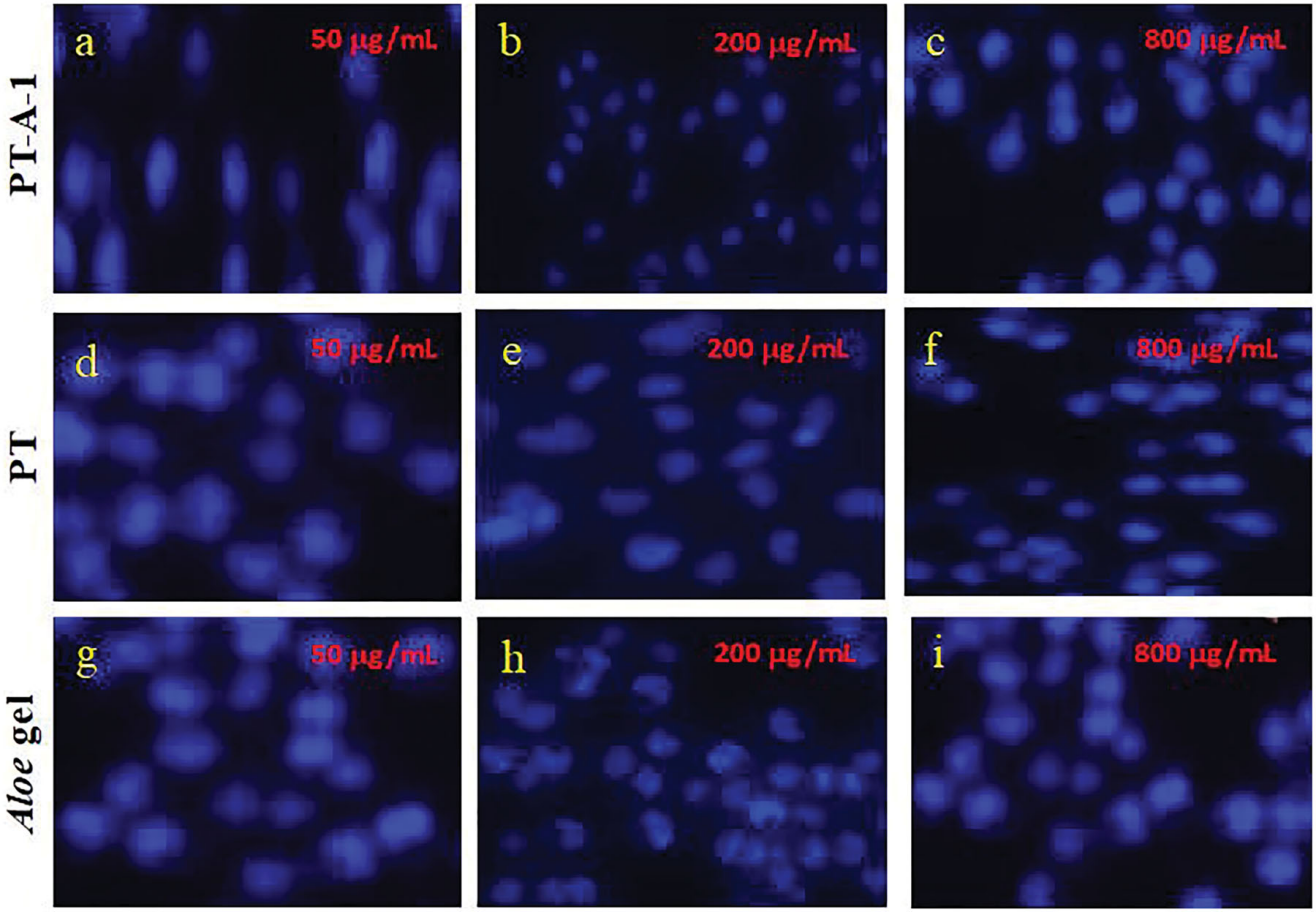
Hoechst 33258 fluorescent dyeing of HaCat cells pre-treated with different concentrations of PT-A, PT, and *Aloe* gel materials for 12 h incubation after 8 Gy X-ray irradiation (200×).

As shown in Figure 3, HaCaT cells were pre-treated with different concentrations of three subgroups (PT-A-1, PT, *Aloe* gel) under optimal experimental conditions, and the irradiated cells were subjected to Hoechst 33258 fluorescence staining at three pre-treatment concentrations per group (in fluorescence staining plots of 50, 200, and 800 μg/mL). The lowest fluorescence intensity was in the 200 μg/mL group, which proved that the apoptosis rate of this group was the lowest. Compared with cells pre-treated with a single material, the cells pre-treated with PT-A-1 had the lowest fluorescence colour, the lowest apoptotic rate, and the highest resistance to ionising radiation. The experimental results indicate that PT-A-1 protects HaCaT cells from ionising radiation. By analysing the fluorescence intensity in the HaCaT cell fluorescence staining diagram, it is clear that the PT-A-1 composite provided optimal protection against ionising radiation when the concentration was 200 μg/mL. The results of this experiment were consistent with the previous CCK-8 test results.

### Flow cytometry for cell apoptosis

**Figure 4.**
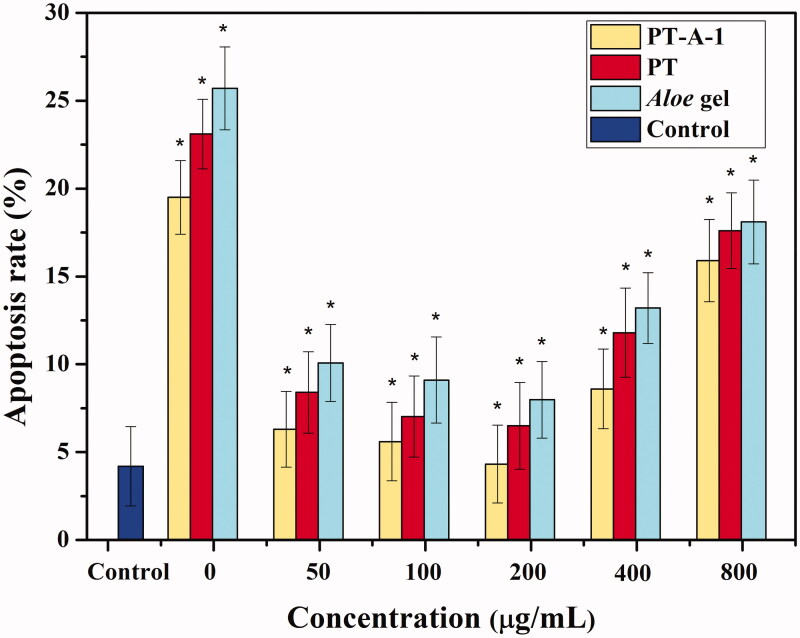
Flow cytometry detecting the apoptosis rate of HaCaT cells pre-treated with different concentrations of PT-A, PT, and *Aloe* gel materials for 12 h incubation after 8 Gy X-ray irradiation. All above values are presented as the median from analysis of three independent experiments and the error bars indicate standard deviation (*n* = 3), Symbol* represents *p* < 0.05 vs. Control group.

As shown in Figure 4, under optimal conditions flow cytometry was used to detect the apoptosis rate of HaCaT cells that were pre-treated with three subgroups (PT-A-1, PT, *Aloe* gel) and irradiated. Among them, the cell apoptosis rate was lowest in the 200 μg/mL concentration group. The HaCaT cells pre-treated with persimmon tannin-*Aloe* gel composite exhibited the lowest apoptosis rate. The flow cytometry results showed that the PT-A-1 can protect HaCaT cells from ionising radiation, and that the apoptosis rate of HaCaT cells was lowest when the concentration was 200 μg/mL. This proved that the PT-A-1 composite provides the strongest protection against ionising radiation at a concentration of 200 μg/mL, which is consistent with the previous CCK-8 activity detection and Hoechst 33258 fluorescence staining results.

### Removal of ROS by persimmon tannin-*Aloe* gel composite

**Figure 5.**
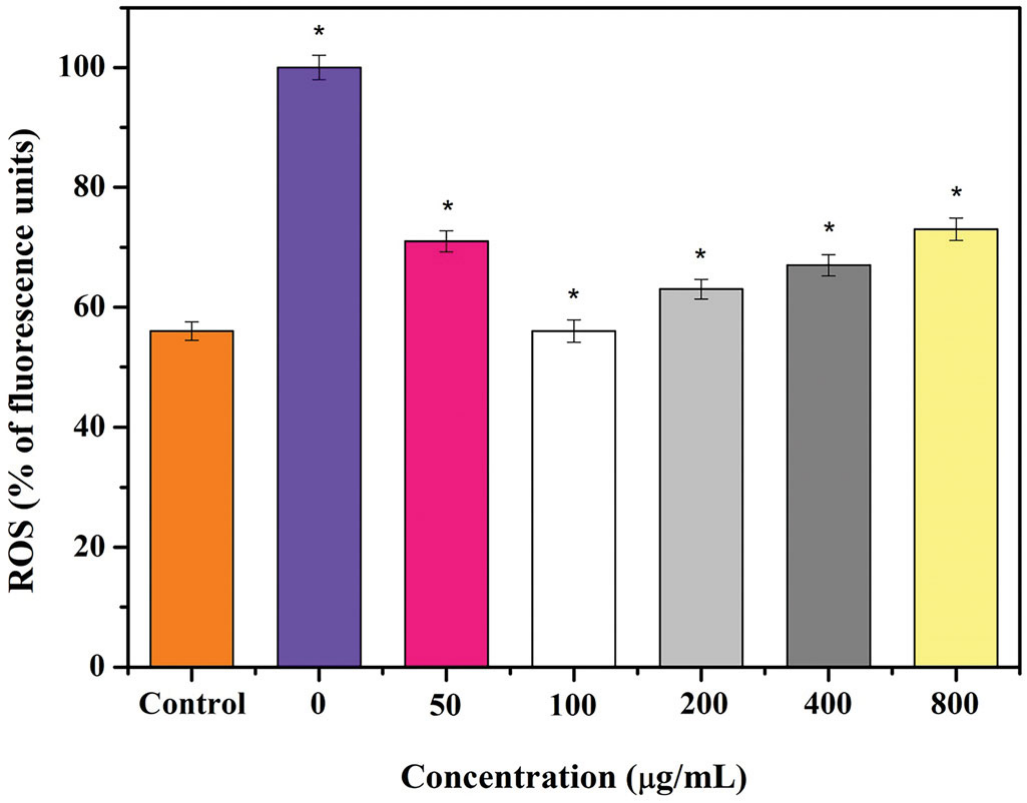
The inhibition effects of PT-A-1 pre-treatment prior to radiation with 8 Gy X-ray irradiation on the production of intracellular reactive oxygen species in HaCat cells. All above values are presented as the median from analysis of three independent experiments and the error bars indicate standard deviation (*n* = 3), Symbol* represents *p* < 0.05 vs. Control group.

Radiation-induced ROS generation was measured in terms of fluorescence using the cell-permeable fluorogenic probe DCFH-DA. The DCFH-DA enters the cell and is easily hydrolysed by intracellular esterases to the nonfluorescent form DCFH, which is rapidly converted to fluorescent DCF in the presence of a variety of ROS. As shown in Figure 5, X-ray doses of 8 Gy resulted in a significant increase in ROS in HaCaT cells compared to the control group. Cells pre-treated with different concentrations (50, 100, 200, 400, and 800 μg/mL) of PT-A-1 composite shows a marked decrease in DCF fluorescence of 69%, 60%, 36%, 55%, and 59% (*p* < 0.05), respectively. Among the pre-treatment concentrations in the experimental groups, the decrease rate of the fluorescence intensity in the 200 μg/mL experimental group was the lowest. Therefore, when the concentration of PT-A-1 composite is 200 μg/mL, it is optimally effective at removing ROS produced by radiation in HaCaT cells and provides the strongest protection against ionising radiation.

### Radiation resistance mechanism

More than half of the substances in an organism are water. Thus, when ionizing radiation illuminates an organism, it can promote the decomposition of water molecules in the cells of the organism, thereby producing a large amount of ROS, which is a leading cause of rapid aging in organisms (Chen et al. 2015). The persimmon tannin-*Aloe* gel composite exerted its radiation protective action through increasing cell viability, reducing cell apoptosis and decreasing the ROS levels of X-ray exposure in HaCaT cells. The results of the above experiments prove that the persimmon tannin-*Aloe* gel composite can provide complete ionizing radiation protection inside and outside the human body.

## Conclusions

This study examined the effectiveness of persimmon tannin-*Aloe* gel composite to protect HaCaT cells from ionizing radiation. X-ray doses of 8 Gy and an incubation time of 12 h after irradiation were deemed to be the optimal experimental conditions. When the mass ratio of persimmon tannin and *Aloe* gel is 2:1 (i.e., PT-A-1), the HaCaT cells exhibited the highest degree of activity and the best radiation resistance with PT-A-1 concentration of 200 μg/mL. In addition, when the concentration of PT-A-1 was 200 μg/mL, the number of apoptotic cells was lowest by Hoechst 33258 dyeing and the lowest apoptotic rate was achieved by flow cytometry assay. The persimmon tannin-*Aloe* gel composite exerts its radiation protective action through increasing cell viability, reducing cell apoptosis and decreasing the ROS levels of X-ray exposure in HaCaT cells. All these results indicated that persimmon tannin-*Aloe* gel composite offered a potent radioprotective effect on cell vitality and cell apoptosis of X-ray exposure in HaCaT cells. This study serves as a pre-clinical evaluation of persimmon tannin-*Aloe* gel composite as a potential natural anti-radiation agent. Future studies will focus on investigating the radioprotective effects of persimmon tannin-*Aloe* gel composite *in vivo* in mice, carrying out the relationship between the structural properties and radioprotective effects of the persimmon tannin-*Aloe* gel composite.
